# Etiopathogenesis of Nonalcoholic Steatohepatitis: Role of Obesity, Insulin Resistance and Mechanisms of Hepatotoxicity

**DOI:** 10.1155/2012/212865

**Published:** 2012-06-25

**Authors:** Praveen Guturu, Andrea Duchini

**Affiliations:** Division of Gastroenterology and Hepatology, University of Texas Medical Branch (UTMB), 301 University Boulevard, Galveston, TX 77555, USA

## Abstract

Incidence of nonalcoholic fatty liver disease is increasing with an estimated prevalence of 20–30% in developed nations. This is leading to increased incidence of chronic liver disease, cirrhosis, and hepatocellular cancer. It is critical to understand the etiology and pathogenesis of any disease to create therapeutic targets and develop new treatments. In this paper we discuss the etiology and pathogenesis of nonalcoholic steatohepatitis with special focus on obesity, role of insulin resistance, and molecular mechanisms of hepatotoxicity.

## 1. Introduction

The term non-alcoholic fatty liver disease (NAFLD) refers to the spectrum of diseases characterized by fatty infiltration of the liver ranging from steatosis, steatohepatitis, or cirrhosis. Hepatic steatosis with or without hepatitis, in the absence of alcohol use, was first described by Ludwig et al. and is referred to as non-alcoholic steatosis or non-alcoholic steatohepatitis (NASH) [1]. NAFLD is a common disease with an estimated prevalence in unselected population of developed nations around 20–30% [2]. The rapid rise in the incidence of the NAFLD might be explained by the recent epidemic of obesity and metabolic syndrome, which are manifested at hepatic level as NAFLD [3–5]. Most patients with NAFLD have simple hepatic steatosis without progression to steatohepatitis and fibrosis. However, in 2-3% of patients, NAFLD can progress to NASH that can eventually cause progressive fibrosis and lead to cirrhosis and related complications including hepatocellular carcinoma [3, 6, 7]. Once patients with simple steatosis develop NASH, up to 50% of them could develop advanced fibrosis [8]. 

A “two-hit hypothesis” was then proposed to explain the pathogenesis and progression of NAFLD, where the first hit causes accumulation of excess triglycerides in the liver leading to simple steatosis and the second hit causes the steatosis to progress to inflammation and fibrosis [9, 10]. The two-hit hypothesis was recently questioned as it was suggested that the hepatic accumulation of triglycerides in the liver might be instead protective towards further hepatic damage [11, 12]. 

Development of obesity or metabolic obesity, defined by isolated increase in visceral fat in people who are not obese, is often seen as the starting point for development of NAFLD [13], leading to the cascade of events ending in the formation of hepatic steatosis. How does increased visceral fat lead to increased fat accumulation in liver? What is the role of insulin resistance? What are the cellular and molecular mechanisms involved? What are the chemical mediators involved? 

## 2. Factors Contributing to Development of Obesity

Development of obesity or metabolic obesity is seen as the initial step in the development of metabolic syndrome and non-alcoholic fatty liver disease. Obesity is likely due to contributions from multiple factors including but not limited to impaired central appetite regulation, genetic predisposition, and contribution from dietary factors and lack of physical activity [13]. 

Central nervous system plays a critical role in regulation of body weight via a negative feedback mechanism. Increase in body fat stores alert the appetite center in hypothalamus leading to appetite control and adipose tissue homeostasis [14]. Insulin and leptin are considered as prime mediators of this mechanism [15]. In overweight individuals, the amount of leptin in circulation is high but they develop resistance to leptin and so their appetite is not well controlled, leading to failure of the negative feedback mechanism [16, 17]. The molecular mechanisms leading to leptin resistance and its role in the development of obesity are discussed in detail elsewhere [18].

While increased caloric intake definitely has a critical role in the development of obesity, there has been considerable interest about various dietary components and their relative contribution to the development of obesity. Increased fructose consumption has been shown as a risk factor for development of NASH and that increased fructose consumption correlates with the severity of fibrosis in patients with NAFLD [19, 20]. The explanation is that fructose consumption leads to obesity or metabolic syndrome and that NAFLD is the hepatic manifestation but it is interesting to note that increased fructose consumption is an independent risk factor for development of fatty liver irrespective of metabolic syndrome [21, 22]. 

Patients with NALFD are shown to have increased percentage of dietary fat content and also get much lower percentage of their calories from fruits [23, 24]; not only total fat content in the diet but also the composition of fat has seen considerable interest in recent times. Current literature supports the fact that diet of patients with NAFLD might be high in saturated fatty acids and n-6 polyunsaturated fatty acids and low in n-3 polyunsaturated fatty acids and monounsaturated fatty acids [25–27]. Though much of the focus has been on diets with high percentage of fats, diet, rich in synthetic disaccharides have also been shown to induce hepatic fibrosis in rats [28].

Gut microbiota and its interaction with the consumed nutrients have also been the focus of research and microbiota could have a possible role in obesity by their influence on amount of nutrients absorbed. Several mechanisms were proposed including altered gut permeability and digesting the ingested polysaccharides thereby increasing the amount of energy absorbed [29].

While low physical activity might not directly contribute to NAFLD in otherwise healthy patients, increase in physical activity coupled with weight loss has been shown to improve liver profile in overweight patients with chronic liver disease [30]. 

## 3. Obesity Is a Proinflammatory State: Results in Insulin Resistance

Two types of adipose tissue are recognized in humans: brown adipose tissue and white adipose tissue. brown adipose tissue, mainly found in neonates, helps with heat production and has a protective effect against hypothermia. White adipose tissue, present in adults, consists of adipocytes, endothelial cells, fibroblasts, leukocytes, and bone marrow derived macrophages. The only function of white adipose tissue was initially thought to be energy store. Instead, new research is pointing towards adipose tissue having a more complex endocrine function mediated by the production of numerous proinflammatory cytokines called adipocytokines [31, 32]. It should also be noted that not all white adipose tissue might be the same; increasing volume of visceral adipose tissue and their production of pro-inflammatory cytokines seems to play an important role in development of insulin resistance compared to subcutaneous adipose tissue [33].

Adipocytokines produced by the adipose tissue include adiponectin, leptin, resistin, visfatin, tumor necrosis factor-*α* (TNF-*α*), interleukin-6 (IL-6), monocyte chemoattractant protein-1 (MCP-1; also known as CCL2 or CC-chemokine ligand 2), plasminogen activator inhibitor-1, angiotensinogen, retinol-binding protein-4, and serum amyloid A [34–38]. Adipocytokines are not exclusively produced by adipocytes but some, like TNF-*α*, are mainly produced by the macrophages in the adipose tissue. MCP-1 produced by adipocytes is a major factor contributing to macrophage recruitment to the adipose tissue [39]. Adipose tissue in obese individuals is associated with increased macrophage activity, which is responsible for almost all of the TNF-*α* and major part of the IL-6 expressed by the adipose tissue [40]. Thus, obesity is state of chronic inflammation characterized by abnormal cytokine (adipocytokines) production and activation of pro-inflammatory signaling pathways [41]. The following sequence of events has been proposed: development of obesity leads to increased volume of adipose tissue, followed by increased production of MCP-1 by the adipocytes, which attracts more macrophages to the adipose tissue itself. Once the macrophages in the adipose tissue are activated, a self-perpetuating inflammatory cascade is triggered by secretion of pro-inflammatory cytokines like TNF-*α* and IL-6 [31]. 

As noted above, the distribution of fat is also important in the pathogenesis of metabolic syndrome and visceral adipose tissue is considered a better indicator of insulin resistance and cardio vascular disease [42]. This could be due to either release of greater amounts of adipocytokines by visceral fat tissue compared to subcutaneous tissue in obese individuals [43] or could be due to the fact that visceral fat has direct access to portal circulation and thereby having stronger impact on liver [34].

Sodium salicylate an antiinflammatory medication has been used to decrease glycosuria associated with diabetes many years before the discovery of association between type-2 diabetes mellitus and increased inflammatory markers [44]. Since then more studies have shown increased levels of inflammatory mediators like C-reactive protein, interleukin-6, and plasminogen activator inhibitor-1 in patients with type-2 diabetes [45–48]. 

Obesity is a proinflammatory state with high levels of circulating pro-inflammatory cytokines and diabetes is also a state of chronic inflammation; how are these two conditions related? The answer to this question was provided by a study that has shown that TNF-*α* can induce insulin resistance in obese rodents and also that neutralization of TNF-*α* can decrease the insulin resistance with resulting increased peripheral uptake of glucose [49]. Since then similar findings of elevated TNF-*α* were also found in humans with increased insulin resistance and impaired glucose tolerance [50–52].

High TNF-*α* levels can induce insulin resistance in animal models through the activation of I-kappa-B-kinase-*β* (IKK*β*)/nuclear-factor-kappa-B (NF-*κ*B) and Jun N-terminal kinase (JNK) pathways [53]. JNK can cause insulin resistance through the phosphorylation of serine residues in insulin receptor substrate-1 (IRS-1) [54, 55]. IKK*β* activation leads to activation of NF-*κ*B via transcription and sub-sequent increased expression of markers and mediators of inflammation causing insulin resistance. Increasing obesity will lead to increased production of adipocytokines like TNF-*α*, IL-6 that lead to perpetuating cycle of JNK, and NF-*κ*B activation leading to worsening insulin resistance. Detailed review of signaling pathways associated with insulin resistance due to inflammation was discussed elsewhere [56]. 

## 4. Mechanisms of Hepatic Fat Accumulation: Role of Insulin Resistance

**Figure 1:**
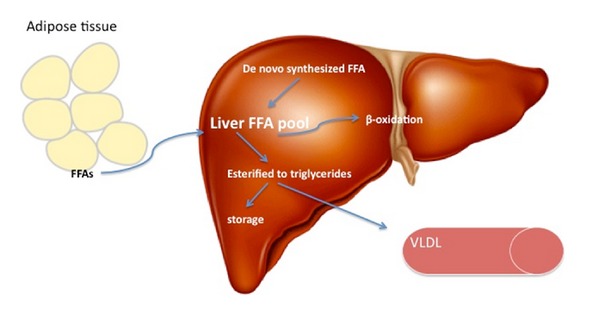
Lipid metabolism in liver. Liver FFA pool is derived from uptake of circulating FFAs and de novo synthesis. The FFAs are then either oxidized or esterified into triglycerides. Triglycerides are then released into circulation as VLDL or stored as vacuoles leading to hepatic steatosis.

The liver plays a key role in lipid metabolism; its role includes uptake and de novo synthesis of free fatty acids (FFAs) followed by conversion of FFAs into triglycerides by esterification. These triglycerides are then released into the circulation as very low-density lipoproteins (VLDL) or stored as triglyceride vacuoles in hepatocytes [57]. FFAs that are not esterified into triglycerides will be metabolized in the liver by *β*-oxidation [58] (Figure 1). 

In NAFLD, there is disruption of this cascade of events since the amount of FFAs delivered/synthesized in the liver exceeds its oxidative capacity. This leads to increased triglyceride synthesis and as the triglyceride synthesis continues to rise and exceed the amount that can be released as VLDLs, triglycerides accumulate in hepatocytes causing hepatic steatosis [58, 59]. This step of development of hepatic steatosis is considered as “first hit” in the pathogenesis of NAFLD [60, 61]. 

This raises the questions: what causes increased availability of FFAs to liver, is it increased delivery or is it due to increased de novo synthesis of FFAs in liver? What is the role of insulin resistance? Other than increased FFA availability, does disruption of other mechanisms like *β*-oxidation or VLDL synthesis contribute to hepatic lipid accumulation? 

As much as 59% of hepatic triglyceride content is derived from free fatty acids and only 26.1% of the hepatic triglyceride was due to de novo synthesis as shown in this study, where isotope tracers were used to track hepatic fat content [62]. This increased delivery of FFAs to liver is due to insulin resistance because insulin resistance increases the total serum FFAs levels due to increased lipolysis in peripheral adipose [63, 64]. Cluster differentiation 36 pathway activation leads to increased FFA uptake by liver [65]. Increased expression of this pathway is seen in patients with insulin resistance and is implicated in pathogenesis of NAFLD [66]. Defective oxidation of the FFAs and dysfunctional VLDL synthesis were also thought to be a key factor in pathogenesis of NAFLD [67]. Though delivery of increased amounts of FFAs beyond the capacity of liver metabolism seems to be the primary cause of hepatic fat accumulation, it should be noted that disruption of other pathways could have a role and more importantly that insulin resistance is implicated in most of these mechanisms [13, 68, 69]. 

As discussed earlier, increased visceral adipose tissue is a risk factor for development of metabolic syndrome and visceral adipose tissue is more prone to insulin resistance when compared to peripheral fat. Insulin resistance in visceral fat leads to increased lipolysis and subsequent delivery of FFAs to the liver increases in an exponential manner due to its direct drainage into portal circulation [70].

## 5. Molecular Mechanisms and Mediators of Hepatotoxicity from Excess Lipids

Hepatic steatosis [61] was considered as first hit in the pathogenesis of NAFLD but it later became clear that accumulation of triglycerides is actually protective and that free fatty acids are the toxic substances that lead to steato-hepatitis and fibrosis [71, 72]. Diacylglycerol acyltransferase 2 (DGAT2) is an enzyme responsible for esterification of FFAs into triglycerides; inhibition of triglyceride synthesis by genetically deleting this enzyme has reduced hepatic steatosis in mouse model but made fibrosis worse due to FFA toxicity [11]. Interruption of triglyceride synthesis could be the initiating event for FFAs-mediated lipotoxicity (cellular toxicity due to accumulated fat) in liver cells [73]. As such, hepatic triglycerides are called the “good fat” and FFAs are called the “bad fat” [74].

This raises the question: are all free fatty acids the same? Studies that looked at the composition of hepatic and circulating free fatty acids have revealed that patients with NAFLD have elevated levels of oleic acid (a monounsaturated fatty acid, MUFA) and palmitic acid (a saturated fatty acid, SFA) [75, 76]. On the other hand polyunsaturated fatty acids are not shown to be toxic to hepatocytes and could be protective in patients with NAFLD [13, 77]. Further information on this topic was provided by experimental studies that looked at the role of stearoyl-CoA desaturase-1 (SCD1), the enzyme that converts SFA to MUFA. Increased expression of SCD1 leads to more MUFA production, which was then incorporated into triglycerides and thus leading to well tolerated simple hepatic steatosis. But inhibition of SCD1 leads to accumulation of SFA and subsequent development of hepatocytes apoptosis and steatohepatitis [74, 78]. So for disease progression in NAFLD, the type of FFA accumulated is as important or may be more important than the quantity of FFAs accumulated in the hepatocytes [79].

Apoptosis is a process of programmed cell death and is considered an important mechanism in the progression of NAFLD [80–82]. Apoptosis is the key pathogenic mechanism noted in the biopsy specimens of the patients with NASH and in the spectrum of NAFLD presence of apoptosis distinguishes patients with simple steatosis from patients with NASH [83]. The extent and severity of the apoptosis correlates with the degree of inflammation and fibrosis, so patients with higher apoptosis rates will have advanced stage fibrosis [80]. Cytokeratin-18 fragments are markers for apoptotic hepatic cells and their circulating levels correlate with the severity of the fibrosis providing further evidence that apoptosis is an important feature of NASH [84]. Apoptosis mediated by FFAs is called lipoapoptosis [85] and the mediators of lipoapoptosis are further discussed here. Apoptotic pathways can be activated via extrinsic pathway mediated by receptors on cell surface or via intrinsic pathway mediated by intracellular organelles [86]. 

### 5.1. Toll-Like Receptors

 Toll-like receptors (TLRs) are pattern recognition receptors that can identify pathogen-associated molecular patterns and in response, they activate the immune system via pro-inflammatory signaling pathways [87]. Saturated fatty acids like palmitic acid can activate TLR4-mediated upregulation of NF-*κ*B with subsequent increased production of adipocytokines like TNF-*α* and IL-6 [88]. Decreased expression of TLR4 in mutant mouse model is shown to be protective against development of NASH [89]. 

In an experimental dextran sulfate sodium (DSS) colitis mouse model, mouse fed with high fat diet and DSS had increased levels of bacterial lipopolysaccharides in portal circulation, increased expression of TLR4, and severe hepatic inflammation when compared to controls [90]. TLR4 might be the crucial link in the gut microbiota-liver axis related to progression of NASH.

### 5.2. Death Receptors

 Death receptors are cell surface receptors from the tumor necrosis factor family of receptors and play critical role in extrinsic apoptotic pathways [91]. The death receptors and their ligands expressed in liver include Fas, tumor necrosis factor receptor 1 (TNF-R1) and TNF-related apoptosis-inducing ligand receptor 1 and 2, TRAIL-R1 and TRAIL-R2, Fas ligand (FasL), TNF-*α*, and TRAIL [92]. In extrinsic pathway, death ligands activate their receptors forming adeath complex that in turn activates caspase-8 leading to apoptosis (caspases are death-inducing proteolytic enzymes). Overexpression of these death receptors and subsequent apoptosis is an important feature of NASH [80].

### 5.3. Mitochondrial Dysfunction and Reactive Oxygen Species 

Reactive oxygen species (ROS) are a group of free radicals derived from molecular oxygen, and oxidative stress refers to the cellular damage done by these free radicals [93]. ROS are formed via oxidative reactions in intracellular organelles and mitochondria are a principal source of ROS, but in a normal healthy cell, the levels of ROS are very low due to various anti-oxidant defense mechanisms [94, 95]. 

In normal healthy subjects, mitochondrial *β*-oxidation is the preferential way to dispose of the FFAs by liver [96]. But in NAFLD, there is an excess of FFAs, and increased *β*-oxidation by mitochondria leads to increased delivery of electrons to the electron transport chain causing overreduction of electron transport chain and formation of ROS [95]. Mitochondrial DNA is vulnerable to damage by ROS; increased generation of ROS leads to damage of mitochondrial DNA leading to mitochondrial dysfunction, which further potentiates ROS formation [97]. 

Intracellular stress caused by accumulation of ROS leads to mitochondrial dysfunction resulting in release of proapoptotic proteins like cytochrome c into the cytosol. Cytochrome c then combines with apoptotic-protein activation factor-1 (Apaf-1) and caspase 9 to form an activation complex called the apoptosome. Apoptosome activates the downstream caspases 3, 6, and 7 to complete the final steps of apoptosis [98]. 

### 5.4. Lysosomal Permeabilization 

Mitochondrial dysfunction is considered the central pathophysiological process contributing to progression of NALFD to NASH, and the quest to identify molecular mechanisms leads to the identification of lysosomal-mitochondrial axis in FFA-induced lipotoxicity and the potential role of lysosomal permeabilization in the progression of NASH [99]. In this study, liver cells were fed with high fat diet and observed in real time, lysosomal permeabilization and cathepsin B (a lysosomal protease) release in the cytoplasm occurred much earlier than mitochondrial dysfunction and cytochrome c release into the cytosol. Also inhibition of cathepsin B was protective against FFA-induced lipotoxicity [99]. Cathepsin B is also implicated in progression of liver fibrosis by its role in activation of hepatic stellate cells and aiding their differentiation into myofibroblasts [100].

### 5.5. Endoplasmic Reticulum Stress

Endoplasmic reticulum (ER) is an intracellular organelle with multiple important functions like protein synthesis, lipid synthesis, and so forth. When ER is put under stress (ER stress), it responds by a mechanism called unfolded protein response (UPR) [101]. UPR is designed to protect ER from the stress induced by various sources like viral infections, alcohol, or FFAs. But when the duration of ER stress is prolonged then UPR might not be able to cope and leads to apoptosis [102, 103]. Further information about the role of ER stress is addressed in this in vitro study where saturated fatty acid palmitic acid was able to induce ER stress and lead to apoptosis of hepatic cells [104]. 

**Figure 2:**
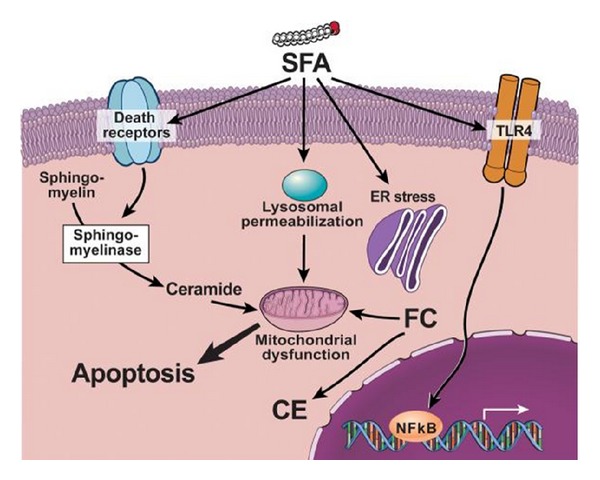
Reference [74]. FFA may activate several signaling pathways of apoptosis including upregulation and increased number of death receptors such as Fas and TRAIL receptor 5 (DR5), at the level of the plasma membrane, lysosomal permeabilization, and ER stress both coupled to mitochondrial dysfunction resulting in activation of the mitochondrial pathway of apoptosis. These toxic fatty acids may also activate TLR4 signaling resulting in up-regulation of several pro-inflammatory cytokines. Finally, other lipid types such as free cholesterol (FC) and ceramide may induce mitochondrial dysfunction and activate the mitochondrial pathway of apoptosis. Abbreviations: FFA: free fatty acids; SFA: saturated fatty acids. MUFA: monounsaturated fatty acids, FC: free cholesterol, CE. cholesteryl-ester; ER: endoplasmic reticulum.

Other mechanisms by which FFAs can lead to apoptosis include mitochondrial dysfunction via c-Jun N-terminal kinase (JNK) activation, pro-apoptotic protein Bax-induced mitochondrial permeabilization, free cholesterol-mediated ER stress, and ceramide-mediated apoptosis induced by death ligands like TNF/FAS [74, 105] (Figure 2). 

**Figure 3:**
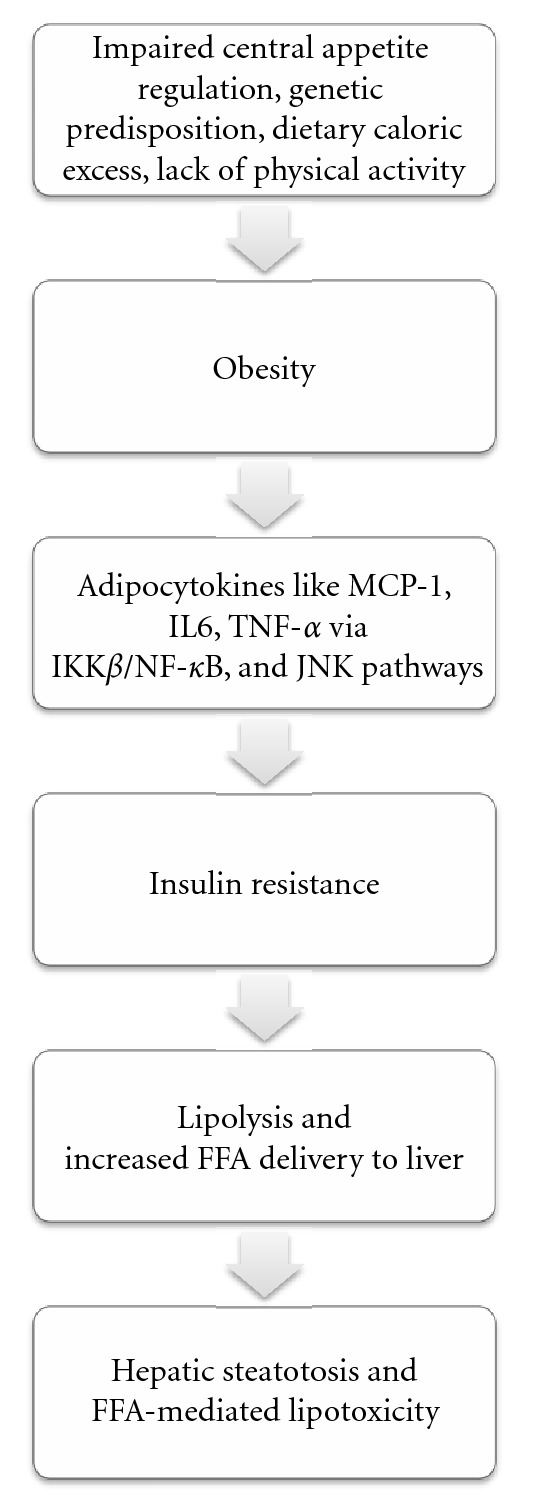
Development and progression of nonalcoholic fatty liver disease.**  **Abbreviations: tumor necrosis factor-*α* (TNF-*α*), interleukin-6 (IL-6), monocyte chemoattractant protein-1 (MCP-1), I-kappa-B-kinase-*β* (IKK*β*), nuclear-factor-kappa-B (NF-*κ*B), Jun N-terminal kinase (JNK), and free fatty acids (FFAs).

*Insummary*, impaired central appetite regulation, genetic predisposition, dietary caloric excess, and lack of physical activity contribute to development of obesity. Obesity is a pro-inflammatory state and leads to insulin resistance via adipocytokines. Insulin resistance leads to increased lipolysis and exponentially high delivery of free fatty acids to liver. Accumulation of FFAs leads to hepatic steatosis and FFA-mediated lipotoxicity that eventually progresses to fibrosis/cirrhosis (Figure 3).

In conclusion, NAFLD is increasing in prevalence and could become the most common cause of chronic liver disease in the near future in the Western world. It is very important to understand the complex molecular mechanisms and the mediator involved to develop new therapeutic targets for this disease.
